# Charismatic Trends in COVID-19 Patients in Pakistan: A Case Series

**DOI:** 10.7759/cureus.19345

**Published:** 2021-11-08

**Authors:** Muhammad Awais Rehan, Amir Waheed, Momin Iqbal, Ali Javed, Shahid R Khalid, Adnan Shabbir

**Affiliations:** 1 Medicine, California Institute of Behavioral Neurosciences & Psychology, Fairfield, USA; 2 Pulmonology Department, Sialkot Medical College, Sialkot, PAK; 3 Molecular Imaging and Neuropathology Department, New York State Psychiatric Institute, New York, USA; 4 Medicine, Abdul Sattar Lab, Sialkot, PAK; 5 Internal Medicine, Sialkot Medical College, Sialkot, PAK; 6 Gastroenterology, Lahore General Hospital, Lahore, PAK

**Keywords:** coronavirus, novel coronavirus-19, severe acute respiratory syndrome, covid-19 pandemic, upper respiratory tract infection

## Abstract

Severe acute respiratory coronavirus-2 syndrome (SARS-CoV-2), the novel coronavirus causing the coronavirus disease (COVID-19), spread across the world, resulting in a global crisis. This pandemic has caused consequences that are beyond the boundaries of a single discipline of life, but it is healthcare that is under the most stress. As we received COVID-19 cases in our hospital (a private tertiary care facility in Sialkot, Pakistan), we geared up to accommodate these cases, since the government sector was already overburdened. The purpose of this study is to report the trends observed in 80 COVID-19 patients admitted at our facility from May 16 to July 14, 2020.

## Introduction

Severe acute respiratory syndrome coronavirus 2 (SARS-CoV-2), the novel coronavirus that caused the COVID-19 pandemic, was first identified in Wuhan (China) [1]. The mode of spread (human-to-human transmission) was described through respiratory droplets, coughing, and sneezing that make the SARS-CoV-2 highly contagious.

Under the influence of this pandemic, cases begin to rise in the local community. Hence, in our hospital (a private tertiary care facility in Sialkot), a team of experts, including a pulmonologist, intensive care specialists, and specialists from diagnostic services, were appointed to manage the crisis. In this study, we have reported the observed trends of our COVID-19 patients that include age and gender distribution, duration of symptoms, the severity of symptoms, mean hospital stay duration, treatment strategies, and overall outcome of the patients. Clinical information was collected by taking a medical history, physical examination, and diagnostic tests. 

## Materials and methods

Our study included patients with COVID-19 infection admitted to our hospital between May 16 to July 14, 2020. Only laboratory-confirmed cases of COVID-19 were included, identified based on a positive result on a reverse-transcriptase-polymerase-chain-reaction (RT-PCR) assay of a specimen collected by a nasopharyngeal swab. Based on it, 80 adults were identified. Further classification of patients was done based on the clinical manifestation of the disease and laboratory and radiological assessments. In our case series, we found an inconclusive impact on severe COVID-19 patients who were treated with tocilizumab [2] or convalescent plasma [3] due to limited sample size.

Clinical specimens for COVID-19 diagnostic testing were collected in compliance with the guidelines of the Ministry of National Health Services Regulations and Coordination (Government of Pakistan). The assay targets the SARS-CoV-2 E gene and RdRp gene [4]. If both parameters are identified, the assay is considered positive; however, if the assay is inconclusive, then we use the reverse-transcriptase-polymerase-chain-reaction (RT-PCR) assay to confirm. The data were summarized using descriptive statistics; categorical variables were listed as percentages and counts. Various sub-groups revealing the statistics of our study, including age, gender, co-morbidities, and mortality pattern, were established. IBM Corp. Released 2013. IBM SPSS Statistics for Windows, Version 22.0. Armonk, NY: IBM Corp. program was used to run analyses.

## Results

We reported 80 patients with confirmed coronavirus (COVID-19) infection in our teaching hospital at Sialkot during the period May 16 to July 14, 2020. This number includes all patients admitted to our hospital under the supervision of our pulmonologist and intensive care team. For better understanding and ease in the interpretation of data, we have sub-divided our data into various sub-groups. Our study population was diverse in multiple aspects. Regarding age and gender distribution, the mean (±SD) age of the patients was 54 (54±15) years; 70% or 56 patients were men. Moreover, for the classification based on the symptomatic manifestation of COVID-19, we used the following parameters. The patients (N=80) were categorized into four groups, i.e., mild, moderate, severe, and critical, according to the characteristics mentioned in Table 1.

**Table 1:** Clinical severity assessment criteria of coronavirus (COVID-19) Patients RT-PCR: real-time reverse transcription–polymerase chain reaction; SARS-CoV-2: severe acute respiratory syndrome coronavirus 2; ARDS: acute respiratory distress syndrome

Mild disease	Moderate disease	Severe disease	Critical illness
Upper respiratory symptoms (e.g., pharyngeal congestion, sore throat, and fever) for a short duration or asymptomatic infection; Positive RT-PCR test for SARS-CoV-2; no abnormal radiographic and septic presentation	Mild pneumonia symptoms such as fever, cough, fatigue, headache, and myalgia; no complications and manifestations related to severe conditions	Mild or moderate clinical features, plus any manifestations that suggest disease progression: rapid breath (≥70 breaths per min for infants aged < 1 year; ≥50 breaths per min for children aged >1 year); hypoxia; lack of consciousness, depression, coma, convulsions, dehydration, difficulty feeding, gastrointestinal dysfunction, myocardial injury; elevated liver enzymes, coagulation dysfunction, rhabdomyolysis, and any other manifestations suggesting injuries to vital organs	Rapid disease progression, plus any other conditions: respiratory failure with need for mechanical ventilation (e.g., acute respiratory distress syndrome [ARDS], persistent hypoxia that cannot be alleviated by inhalation through nasal catheters or masks) septic shock, organ failure that needs monitoring in the ICU

**Figure 1:**
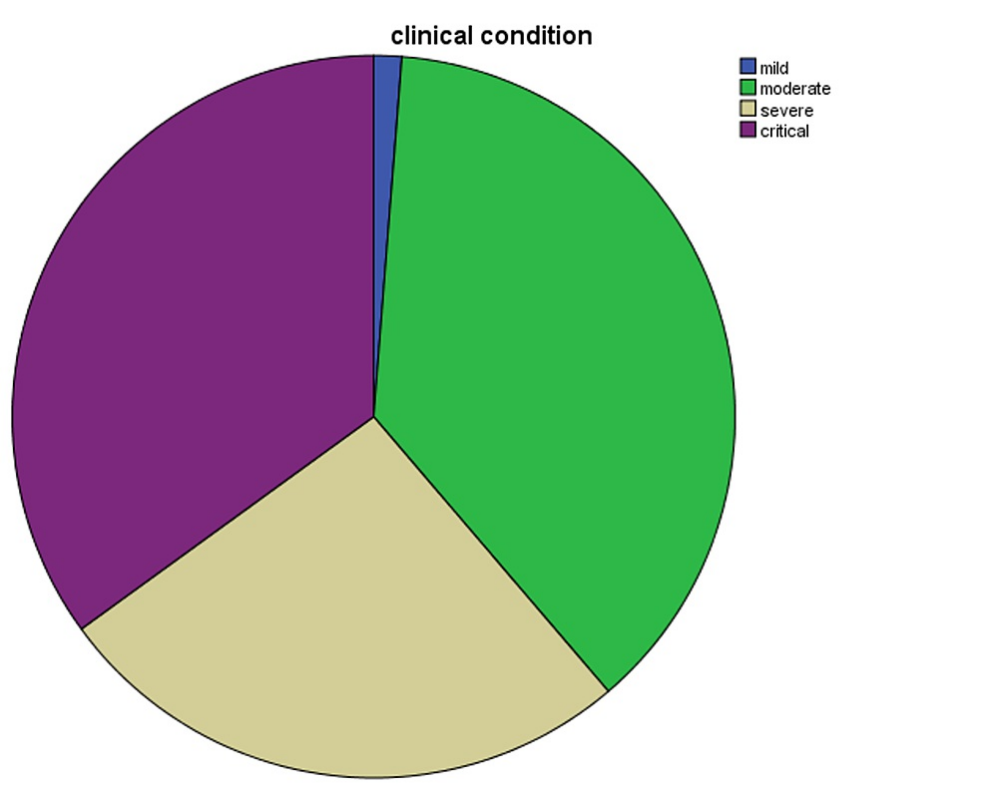
Distribution of coronavirus (COVID-19) patients according to clinical severity Total patients = 80; patients with mild symptoms = two (2%), patients with moderate symptoms = 30 (37%), patients with severe symptoms = 20 (26%) and patients with critical symptoms = 28 (35%).

Once the categorizing based on symptomatic manifestation was done, we got interesting results that include an overall estimation regarding the duration of the onset of symptoms and a percentage of the population with its severity of the disease. The duration of manifestation of symptoms began seven (7±3) days before admission, with the shortest duration being four days after onset of symptoms to get admitted to our hospital, and the longest duration of the onset of symptoms is 10 days. Cough and fever were the most common symptoms among all the clinical symptoms and parameters mentioned in Table 1. The patients (N=80) were categorized into four groups, i.e., mild = two patients (2%), moderate = 30 patients (37%), severe = 20 (26%) and critical = 28 (35%), as shown in Figure 1.

A detailed history and examination of the patients revealed that some of the patients have other relevant factors influencing the overall outcome of the patients such as underlying comorbid conditions like hypertension (HTN), diabetes (DM), ischemic heart disease (IHD), chronic kidney disease (CKD), and asthma. Out of 80 admitted patients, 34 patients (42%) didn't have any associated comorbid conditions whereas 16 patients (20%) were hypertensive (HTN), 14 patients (17.5%) were diabetic (DM), four patients (5%) were ischemic heart disease patients (IHD), two patients (2.5%) were diagnosed with chronic kidney disease (CKD) whereas one patient (1%) was asthmatic, as elaborated in Table 2.

**Table 2:** Comorbidities among patients DM: diabetes; HTN: hypertension); CKD: chronic kidney disease; IHD: ischemic heart disease; HCV: hepatitis C infection; CVA: cerebrovascular accident

		Frequency	Percent
Valid	None	34	42.5
	Diabetes (DM)	5	6.3
	Hypertension (HTN)	13	16.3
	Chronic kidney disease (CKD)	1	1.3
	Diabetes and hypertension (DM+HTN)	10	12.5
	Diabetes and ischemic heart disease (DM+IHD)	2	2.5
	Diabetes, hypertension, and ischemic heart disease (DM+HTN+IHD)	4	5.0
	Diabetes, hypertension, ischemic heart disease, chronic kidney disease (DM+HTN+IHD+CKD)	1	1.3
	Hepatitis C infection (HCV)	1	1.3
	Diabetes, hypertension, and hepatitis C infection (DM+HTN+ HCV)	1	1.3
	Asthma	3	3.8
	Diabetes, hypertension, ischemic heart disease, and hepatitis C infection (DM+HTN+IHD+ HCV)	1	1.3
	Rheumatoid arthritis+B-cell lymphoma	1	1.3
	hepatitis C infection (HCV) and Mysthenia gravis	1	1.3
	Hypertension, and ischemic heart disease (HTN, IHD)	1	1.3
	Diabetes, cerebrovascular accident (DM, CVA)	1	1.3
	Total	80	100.0

In our study, the age distribution of COVID-19 infected patients revealed that COVID-19 infection was more prevalent among those who were over 50 years. Moreover, the recovery from COVID-19 infection was noticed to be bleaker with an increase of age (beyond 60 years). Among the age group of 10-49, the average survival rate was 60%, whereas, among the age group beyond 49 years (49-89), the average survival rate was 35 %. Detailed analysis of recovery and deaths according to age is shown in Table 3.

**Table 3:** Analysis of recovery and deaths according to age

Age group	Fully recovered	Died	Total individuals
10-19	2 (100%)	0 (0%)	2
20-29	1 (50%)	1 (50%)	2
30-39	9 (81%)	2 (19%)	11
40-49	9 (75%)	3 (25%)	12
50-59	18 (75%)	6 (25%)	24
60-69	7 (35%)	13(65%)	20
70-79	2 (33%)	4 (66%)	6
80-89	1 (33%)	2 (66%)	3
Total	49(61%)	31(39%)	80

In the next step, we compared the results of different treatment options administered to the patients that include: ventilatory support, continuous positive airway pressure therapy (CPAP), bilevel positive airway pressure (BiPAP), tocilizumab [2], and convalescent plasma [3]. Out of a total of 80 patients, 28 patients (35%) required mechanical ventilation [4], 26 patients (32.5%) required CPAP, and five patients (6.3%) required BiPAP. Of the 28 patients who received invasive mechanical ventilation, three patients (10%) had been successfully extubated, whereas the rest of the 25 patients (90%) died.

Of the total 80 patients, 16 (20%) patients were administered tocilizumab [2]. Regardless of severity, out of 16 patients who were given tocilizumab, eight (50%) patients recovered successfully, while the remaining eight patients (50%) died. Moreover, out of 80 patients, six patients received convalescent plasma [3]. Among those who received convalescent plasma, four patients (67%) expired, whereas two patients (33%) successfully recovered. The patients in our series presented with respiratory symptoms similar to those of patients described in the literature [5], which indicates a common host response to SARS-CoV-2.

**Figure 2:**
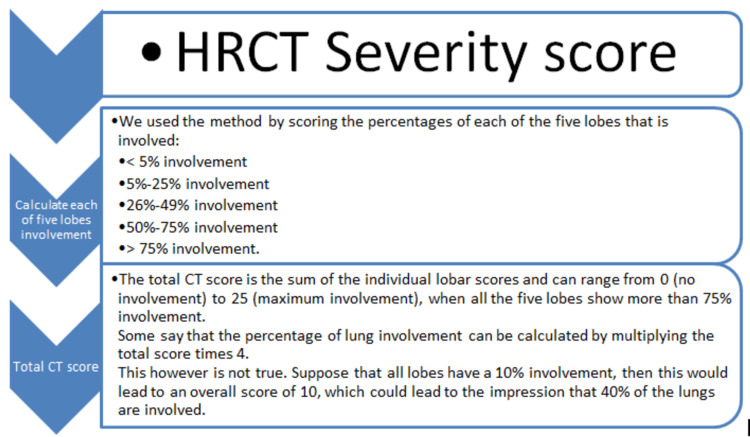
High-resolution computed tomography (HRCT) severity score HRCT: high-resolution computed tomography; CT: computed tomography scans

Furthermore, we elaborated the pattern of variation in the laboratory and radiologic modalities that include chest computed tomographic (CT) and C-reactive protein (CRP), serum ferritin, D-dimers, creatinine kinase, interleukin-6, and procalcitonin levels. A chest CT scan was obtained in 80 patients (100%) on ICU admission; the scans showed variable presentations comprising bilateral ground-glass opacities, crazy paving, vascular dilatation, traction bronchiectasis, subpleural bands, and architectural distortion [6]. The high-resolution CT (HRCT)-chest scoring system [7] used at the time of admission and on follow-up at two weeks is depicted in Figure 2.

Regarding the mean C-reactive protein (CRP) [8] value (of all 80 patients) was 104, mean value of serum ferritin [9] was 4176, mean value of D-dimers [10] was 1016, mean value of creatinine kinase [11] was 148, mean value of interleukin-6 [12] was 428, mean value of procalcitonin [13] was 19, while the mean value of lactate dehydrogenase (LDH) [14] was 556, as elaborated in Table 4.

**Table 4:** Laboratory tests pattern among COVID-19 patients CRP: C-reactive protein; S. Ferritin: serum ferritin; LDH: lactate dehydrogenase

		CRP	S. Ferritin	D-dimers	Creatinine Kinase	Interleukin 6	Procalcitonin	LDH
Mean		104.47	4176.61	1016.26	148.07	428.15	19.06	556.41
Std. Deviation		107.87	1536.40	1036.21	133.06	1006.13	34.85	297.91

Regarding the outcome of the patients, from the total of 80 patients, 32 patients (40%) died. Of the 48 surviving patients, 41 patients (85%) were discharged home with full recovery from symptoms, while seven patients (15%) were discharged home on oxygen therapy.

## Discussion

SARS-CoV-2, the novel coronavirus, spread rapidly and resulted in mild to critical illness. Scientific analyses of virus RNA from patients infected in Sialkot revealed that SARS-CoV-2 was the causative agent. Our study aims to elaborate on clinical features and outcomes among COVID-19-infected patients in Sialkot.

This case series describes 80 patients at our teaching hospital, Sialkot, from May 16 to July 14, 2020. All patients with documented exposure had acquired COVID-19. Some of the patients had co-morbidities like diabetes mellitus, hypertension, and chronic kidney disease. Though in individuals 55 years of age or older, the fatality rate was higher. Of the 28 patients, who received intrusive mechanical ventilation, three were successfully extubated. Our study has several limitations, including incomplete documentation of clinical features and laboratory investigations. Additionally, our sample size is limited due to our focus on patient care at the time of the pandemic. Specifically, this involves patients on the general medical ward who had provided treatment focusing solely on comfort measures.

The early COVID-19 pandemic results showed similar trends in Pakistan as was seen in other countries, including mortality rates, especially in patients with co-morbidities. With such patients, more information is required to improve the treatment strategies. Our results also illustrate the increased demand for hospital care for the treatment of COVID-19 patients in Pakistan.

In our case series, we found an inconclusive impact on severe COVID-19 patients who were treated with tocilizumab [2] or convalescent plasma [3] due to limited sample size. Regarding the outcome of the patients, from the total of 80 patients, 32 patients (40%) died. Of the 48 surviving patients, 41 patients (85%) were discharged home with full recovery from symptoms, whereas seven were discharged on oxygen.

## Conclusions

During the COVID-19 outbreak in Sialkot (Pakistan), the patients with severe and critical categories of COVID-19 symptoms showed markedly increased duration of hospital stays and mortality. For a detailed study, we have categorized our data into various subgroups based on age, gender, clinical symptoms, laboratory parameters, radiological findings, management strategies, and terminal outcomes. Regarding the outcome of the patients, from the total of 80 patients, 32 patients (40%) died. Of the 48 surviving patients, 41 patients (85%) were discharged home with full recovery from symptoms, while seven patients (15%) were discharged home on oxygen therapy.
